# Wide angled ‘V’ is the perfect disposition of a TIVAD catheter when right internal jugular vein is cannulated to gain central access

**DOI:** 10.1093/gastro/goz027

**Published:** 2019-07-15

**Authors:** Chitresh Kumar, Chandan Kumar Jha, Raouef Ahmed Bichoo, Sanjay Kumar Yadav

**Affiliations:** 1 Department of Endocrine Surgery, Sanjay Gandhi Postgraduate Institute of Medical Sciences, Lucknow, India; 2 Department of Surgical Oncology, All India Institute of Medical Sciences, New Delhi, India; 3 Department of General Surgery, All India Institute of Medical Sciences, Patna, India

## Introduction

A TIVAD (totally implantable venous access device) catheter is inserted into one of the large veins, usually the superior vena cava (SVC), to gain permanent central venous access. The tip of the catheter sits just above the heart in the distal third of the SVC and the other end is attached to the silicon rubber port that sits underneath the skin of the anterior chest wall fixed to the fascia of the pectoralis major muscle at the lateral part of the second rib [1]. When the access is made through the right internal jugular vein (IJV), complications are fewer compared to cannulation of the left IJV or subclavian veins [2, 3]. One of the issues that is frequently encountered during catheter placement in the right IJV is the kinking of the catheter, leading to compromised or absent flow despite successful cannulation and positioning of the central venous catheter.

## Learning point

**Figure 1.**
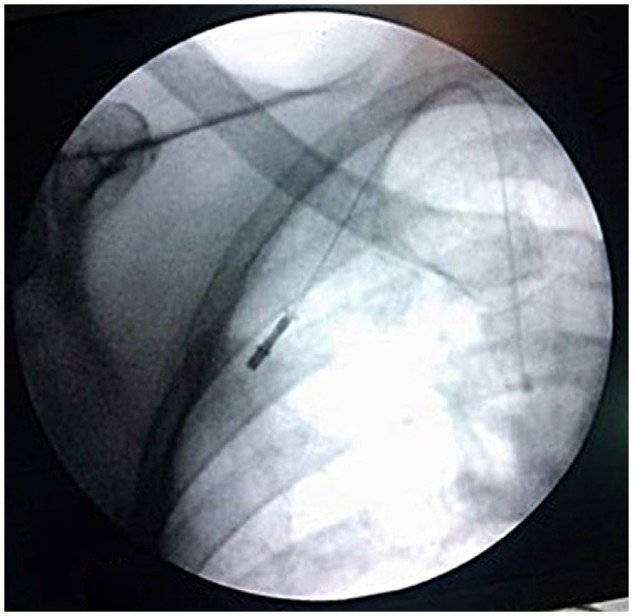
Intra-operative picture depicting the proper disposition of the catheter

The proper position of the catheter before fixing it to the port is of paramount importance to avoid complications. One of the most commonly encountered intra-operative complications when accessing the right IJV for central venous access is the kinking of the catheter, either at the site of catheter entering into the IJV or at the site of catheter joining the port. Both of these kinks can be avoided if one aims for a disposition of the venous catheter as depicted in Figure1 by minor modifications in the operative technique (Figure 1). On fluoroscopy, the catheter should look like an inverted ‘V’, with a wide angle at the apex of the ‘V’ that should be located in the supra-clavicular fossa. The tip of the catheter should lie just above the right atrium and the port should be in the second intercostal space in the lateral part.

## Conflicts of interest

None declared.
